# Incidence and risk factors of retreatment after three-monthly aflibercept therapy for exudative age-related macular degeneration

**DOI:** 10.1038/srep44020

**Published:** 2017-03-07

**Authors:** Wataru Kikushima, Yoichi Sakurada, Seigo Yoneyama, Atsushi Sugiyama, Naohiko Tanabe, Atsuki Kume, Fumihiko Mabuchi, Hiroyuki Iijima

**Affiliations:** 1 Department of Ophthalmology, Yamanashi University Chuo Yamanashi, Japan

## Abstract

Though anti-vascular endothelial growth factor therapy has become the standard treatment for exudative age-related macular degeneration (AMD), retreatment after the initial loading injection is inevitable in most eyes with residual or recurrent exudative changes. In the present study, we studied 140 treatment naïve eyes with typical neovascular AMD (n = 71) or polypoidal choroidal vasculopathy (PCV) (n = 69) and investigated the incidence and risk factors of retreatment after 3-monthly intravitreal aflibercept injection for exudative AMD during the 12-month period. At 12 months, best-corrected visual acuity (BCVA) improved significantly from 0.45 ± 0.39 to 0.26 ± 0.33 (P = 4.1 × 10^−11^). Multiple regression analysis revealed that better baseline BCVA (P = 3.6 × 10^−14^) and thicker subfoveal choroidal thickness (P = 0.039) were associated with better BCVA at 12-months. Retreatment was required in 94 out of 140 (67.1%) eyes. Multivariate logistic regression analysis revealed that older age (P = 7.2 × 10^−3^) and T-allele of *ARMS2* A69S (rs10490924) variants (P = 1.9 × 10^−3^) were associated with retreatment. Cox-regression analysis revealed that older age (P = 1.0 × 10^−2^) and T-allele of the *ARMS2* gene (P = 6.0 × 10^−3^) were associated with retreatment-free period. The number of retreatment episodes was significantly different among the *ARMS2* genotypes (P = 8.1 × 10^−4^). These findings might be helpful for physicians when considering the optimal treatment regimen for exudative AMD.

Age-related macular degeneration (AMD) is one of the leading causes of blindness in the industrialized countries^1^,^2^. It has been proven that vascular endothelial growth factor (VEGF) is a crucial factor in stimulating the development of exudative AMD^3^. The management of exudative AMD has been revolutionized by the introduction of anti-VEGF agents. While intravitreal injection therapy with anti-VEGF agents has become the standard treatment for exudative AMD worldwide, retreatment after the initial loading injection is inevitable in most eyes with residual or recurrent exudative changes, including hemorrhage and intraretinal or subretinal fluid. Many clinical trials have challenged several retreatment regimens, e.g. fixed-interval regimen^4^,^5^,^6^,^7^,^8^,^9^,^10^; treat and extend regimen (TAE)^2^,^11^, and the *pro re nata* (PRN) regimen^8^,^9^,^10^,^11^; however there have been controversies among retinal specialists regarding the optimal regimen.

Fixed-interval regimen and TAE are referred to as proactive treatments, which aim to maintain the integrity of the photoreceptors by intravitreal injection of anti-VEGF agents before the recurrence of exudative changes. However, these are theoretically endless treatment modalities that could lead to the potential for overtreatment, because patients must receive intravitreal injections forever, regardless of the presence or absence of choroidal neovascularization.

In the PRN regimen, patients cease treatment if the subretinal pathology, such as choroidal neovascularization, becomes inactive. This approach reduces the risk of both ocular and systemic complications, as well as medical expenses. HARBOR Study Group demonstrated that three monthly intravitreal ranibizumab followed by monthly-monitoring and as-needed retreatment was equivalent as monthly intravitreal ranibizumab, with 8.2 and 10.1 letters gained at 12 month, respectively^9^.

Aflibercept is the most recently approved intravitreal anti-VEGF agents. The *V*egf-trap eye *I*nvestigation of *E*fficacy and safety in *W*et AMD (VIEW) 1 and 2 studies revealed that bimonthly intravitreal injection of 2.0 mg aflibercept after a loading phase of three-monthly injections resulted in similar visual outcomes to monthly intravitreal injection of 0.5 mg ranibizumab^6^. Several subsequent reports have demonstrated that visual outcomes were favorable using similar fixed-interval regimens with aflibercept^12^,^13^,^14^.

In the present study, we retrospectively studied the efficacy of the PRN regimen for aflibercept after the 3-monthly loading injections for exudative AMD and investigated the 12-month visual outcome and incidence of retreatment as well as their respective risk factors.

## Results

During the study period, we treated 156 eyes from 172 patients, however 16 patients (10.3%) could not complete 12-month follow-up. Therefore we included 146 eyes from 140 patients in this study. Because only the second treated eye was included in the present study for each eligible patient with bilateral involvement, a total of 140 eyes from 140 patients were investigated. Table 1 presents the demographic and genetic characteristics of all of the subject patients, consisting of typical neovascular AMD (n = 71) and PCV (n = 69). While patients with typical neovascular AMD were significantly older with a greater baseline CRT, a higher risk allele frequency of the *ARMS2* A69S (rs10490924) and the *CFH* I62V (rs800292) variants and worsened baseline BCVA compared with those with PCV, BCVA improvement and reduction of central macular thickness and subfoveal choroidal thickness were similar among both subtypes at the 3-monthly visits during the 12-month follow-up period (Table 2).

Table 3 presents the results of multiple regression analyses to examine the factors associated with BCVA and visual gain at 12 months. Both better baseline BCVA and thicker baseline subfoveal choroidal thickness were associated with better BCVA at 12 months.

Retreatment of single or multiple additional IAI was required after the initial 3-monthly IAI in 94 out of 140 eyes (67.1%) during the 12-month follow-up period. Table 4 presents the clinical and genetic characteristics of patients with or without retreatment and the results of univariate and multivariate logistic regression analyses associated with retreatment during 12-month follow-up period. While patients requiring retreatment were significantly older (P = 3.1 × 10^−3^, chi-square test) with thinner subfoveal choroids (P = 0.036, Mann Whitney U test), longer GLD (P = 0.049, Mann Whitney U test) and higher T-allele frequencies of the *ARMS2* A69S (rs10490924) (P = 2.7 × 10^−4^, chi-square test) in univariate analysis, the association with subfoveal choroidal thickness and GLD was eliminated in multivariate logistic regression analysis, which revealed that older age (Odds ratio: 1.08, 95% confidence interval [95% CI]: 1.02–1.14, P = 7.2 × 10^−3^, multivariate logistic regression analysis) and T-allele of *ARMS2* A69S (rs10490924) variants (Odds ratio: 2.46, 95% CI: 1.39–4.35, P = 1.9 × 10^−3^, multivariate logistic regression analysis) were associated with retreatment. In each subtype analysis, patients without requiring retreatment were tend to be younger with less T allele of *ARMS2* A69S compared with those requiring retreatment though a statistical significance was only seen in T allele frequency of *ARMS2* A69S between PCV patients with or without retreatment (P = 0.02, multivariate logistic regression analysis).

**Figure 1:**
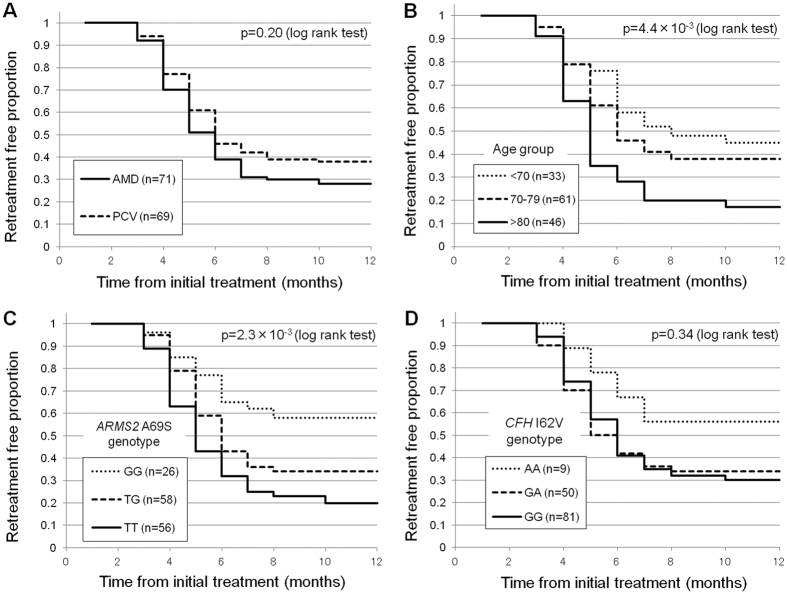
Kaplan-Meier plots showing retreatment-free proportion by AMD subtypes (**A**), age groups (**B**), ARMS2 A69S genotypes (**C**) and CFHI62V genotypes (**D**).

Figure 1 presents the Kaplan-Meier curves of the retreatment-free period according to the AMD subtypes, age groups, *ARMS2* A69S genotypes and *CFH* I62V genotypes. The mean retreatment free period was significantly longer in non-carriers of the *ARMS2* A69S T-allele genotype (9.15 months, 95% CI: 7.83–10.48) than in those that were heterozygous (7.50 months, 95% CI: 6.62–8.38) and homozygous (6.38 months, 95% CI: 5.55–7.20) for the T-allele (p = 2.3 × 10^−3^, log-rank test). Cox regression analysis revealed that older age (Hazard ratio: 1.04, 95% CI; 1.01–1.07, P = 1.0 × 10^−2^) and T-allele of *ARMS2* A69S (Hazard ratio: 1.53, 95% CI; 1.13–2.07, P = 6.0 × 10^−3^) were associated with the retreatment-free period (Table 5).

**Figure 2:**
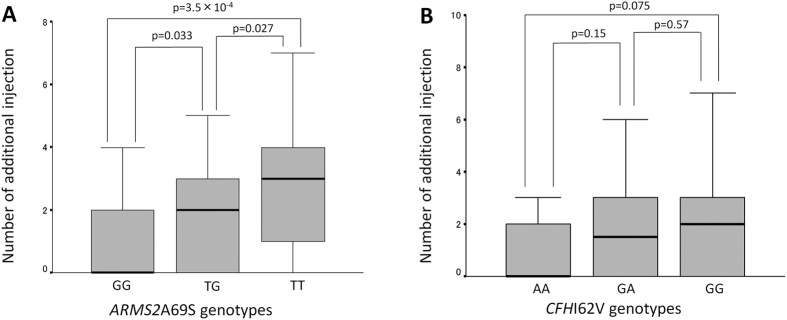
Number of additional injections with intravitreal aflibercept stratified by the *ARMS2* A69S (**A**) and *CFH I62V* genotypes (**B**).

Figure 2 presents the number of additional injections of retreatment based upon the *ARMS2* A69S and *CFH* I62V genotypes. The median number of additional injections was 0, 2, and 3 for the GG, TG, and TT of *ARMS2* A69S genotypes, respectively (P = 8.1 × 10^−4^, Kruskal-Wallis test), compared with 0, 1.5, and 2 in AA, GA, and GG of *CFH* I62V genotypes, respectively (P = 0.19, Kruskal-Wallis test).

Table 6 presents the change of BCVA in patients with or without retreatment. While the difference of visual gain between eyes with and without retreatment tended to get larger as the follow-up period was prolonged, the difference at 12 months was not significant.

## Discussion

In the present study, we studied the incidence of retreatment of IAI with the PRN regimen after a loading dose of 3-monthly IAI in 140 eyes of 140 patients with exudative AMD as well as the visual outcome.

Ninety-four eyes (67.1%) required retreatment of additional single or multiple IAIs including the initial 3-monthly IAI treatments during the 12-month follow-up period due to residual or recurrent exudative changes in the macula. Univariate analyses revealed that retreatment was associated with thinner subfoveal choroidal thickness, longer GLD, older age and T-allele of *ARMS2* A69S (rs10490924) genetic variants (Table 4).

Because the phenotypic features of exudative AMD, i.e., subfoveal choroidal thickness, and lesion size have been reported to be associated with genetic factors, including *ARMS2* and *CFH*^15^,^16^,^17^,^18^, we conducted multivariate logistic regression analyses, which revealed that only older age and the T-allele of *ARMS2* A69S (rs10490924) were associated with retreatment.

Recently Kuroda *et al*.^19^ conducted a similar retrospective cohort study to investigate the factors requiring PRN retreatment with IRI in 236 eyes with neovascular AMD, who received initial loading dose of 3-monthly IRIs and exhibited no exudative changes (dry macula) one month after the third injection of the planned IRIs. The results, however, demonstrated that older age and male patient were associated and that the *ARMS2* A69S genotype was not associated with retreatment for additional IRI. The major differences between the two studies of Kuroda’s and ours include different anti-VEGF agents (ranibizumab VS aflibercept), proportion of treatment-naïve patients (77% VS 100%) and the definition of retreatment (i.e., additional injection for reappearance of exudative changes after the initial resolution of it after 3-monthly loading treatment VS any additional injections regardless of the resolution of exudation after 3-monthly loading treatment). We included additional injection for residual exudation in eyes not achieving dry macula after 3-monthly loading treatments because one of our aims was to study the completeness of the initial 3-monthly injections.

While both studies had some differences, i.e., inclusion criteria and definition of retreatment, the *ARMS2* A69S genotype was greatly associated with the additional treatment in the present study (OR: 2.46, 95% CI: 1.39–4.35), while its association with retreatment for recurrence after achieving dry macula was insignificant in the report by Kuroda *et al*. Further investigation of these differences is necessary.

In the present study, log MAR BCVA improved significantly from 0.45 to 0.26 at 12 months, corresponding to a visual gain of 9.5 ETDRS letters, which is equivalent to the visual gain reported in the previous multicenter trials, including the VIEW 1 and 2 studies. Multivariate regression analysis revealed that better BCVA at 12 months was associated with better baseline BCVA and thicker subfoveal choroid. The present study could not demonstrate any effect of genetic variants including *ARMS2* and *CFH* on the visual gain after 3-monthly IAIs followed by retreatment of the IAI with the PRN regimen.

In the literature, there have been conflicting results regarding the relationship between genetic variants and visual gain after intravitreal injections of anti-VEGF agents. Some have reported associations with the *ARMS2* genotype^20^,^21^ and or *CFH* genotype^21^,^22^,^23^ and others denied the association^24^,^25^,^26^.

Although no consistent conclusions were obtained regarding the relationship between genetic variants and visual gain after intravitreal injections of anti-VEGF agents, previous studies demonstrated the relationship between genetic factors and the treatment outcomes, including visual outcome and recurrence after photodynamic therapy (PDT) for exudative AMD in Japanese^27^,^28^,^29^. The non-risk G allele of *ARMS2* A69S (rs10490924) was associated with a significantly better BCVA at 12 months after the first PDT in both neovascular AMD and PCV patients^27^,^28^,^29^. While higher-risk genotypes of *ARMS2* or *HTRA1* gene was significantly associated with recurrences after PDT leading to severe vision loss, than lower-risk genotypes^27^,^28^, it remains to be proved whether visual outcome is deteriorated due to liability to recurrence after IAI in eyes with risk variants of *ARMS2*, which has been demonstrated in the present study.

Although the visual gain at 12 months was not different between eyes with and without requiring retreatment in this study, the mean difference of log MAR BCVA is getting greater in the post-treatment course showing 0.01, 0.03, 0.05 and 0.07 at 3-, 6-, 9, and12-months from the initial injection (Table 6). It may indicate that the eyes with recurrence of exudative changes finally result in poorer visual outcome due to accumulated photoreceptor damages induced by recurrence although the statistically significance of the different BCVA was not demonstrated at 12 months.

Consistent with Kuroda’s results the present results demonstrated that older age is significantly associated with retreatment. It may indicate that younger patients with exudative AMD may be expected to receive additional injections less frequently after 3-monthly IAI treatments than in elderly patients. Therefore PRN regimen may be more appropriate after 3-monthly IAI treatments for such younger patients. On the other hand, elderly patients tend to require additional injections more frequently after 3-monthly IAI treatments. Therefore TAE instead of PRN might be more appropriate for such elderly patients. Considering age and genotyping of the *ARMS2* variant the chance and the timing of retreatment may be speculated, which might be helpful to consider which regimen is more appropriate for the patient between PRN or TAE after 3-monthly IAI treatment. It might be worthwhile selecting either PRN or TAE regimen after 3-monthly IAI treatment in terms of reducing medical burden, cost and risks connecting intravitreal injections.

There are several limitations in this study. The major limitation is a retrospective analysis in this study. The second limitation of study is that we did not consider fluid-related and vitreo-macular interface features in this study. The third limitation of study is that BCVA was examined using Landolt C chart instead of ETDRS chart. The fourth limitation is a relatively small sample size. To confirm the tentative conclusion, it will be necessary to perform a large cohort prospective study. We genotyped only two major genetic variants susceptible to AMD, *ARMS2* and *CFH*. Further genetic analysis will be necessary to investigate the association between genetic factors and IAI treatment.

In conclusion, visual outcomes after 3-monthly IAI followed by PRN regimen were favorable irrespective of AMD subtypes over 12 month. Retreatment was associated with older age and T-allele of the *ARMS2* A69S genotypes. These findings might be helpful for physicians to select the treatment regimen for exudative AMD.

## Methods

### Subjects

A retrospective medical chart review was conducted in consecutive eyes with treatment naïve subfoveal exudative AMD receiving 3-monthly intravitreal aflibercept injections (IAIs) at the University of Yamanashi Hospital between January 2013 and July 2015. The study was approved by the institutional review board at the University of Yamanashi and adhered to the tenets of Declaration of Helsinki. Written informed consent was obtained from each patient before treatment.

All patients had undergone comprehensive ophthalmic examination, including measurement of BCVA, using Landolt chart and intraocular pressure (IOP), slit-lamp biomicroscopy with +78-diopter (D) lens, color fundus photography, fluorescein and indocyanine green angiography using a confocal laser scanning system (HRA-2; Heidelberg Engineering, Dossenheim, Germany), and spectral domain optical coherence tomography (SD-OCT) examination (Spectralis version 5.4 HRA + OCT).

The greatest linear dimension (GLD), which was defined as the fundus lesion covering the dye-leaking, pigment epithelial detachment, subretinal hemorrhage, and choroidal neovascularization, if present, was determined in the fluorescein angiography image. The central macular thickness was defined as the vertical distance between the inner border of the retinal pigment epithelium and the inner limiting membrane at the center of the macula in the SD-OCT image. The subfoveal choroidal thickness was measured as the vertical distance between the outer border of the retinal pigment epithelium and the chorioscleral border, using the enhanced depth-imaging mode equipped with HRA2 Spectralis ver 5.4.

The inclusion criteria were (1) eyes with treatment-naïve AMD, including typical neovascular AMD and polypoidal choroidal vasculopathy; (2) baseline decimal BCVA ≤ 1.2 in the Landolt chart, (3) receiving three-monthly intravitreal aflibercept (2.0 mg/0.05 ml); and (4) a minimum follow-up period of 12 months after the initial injection. The exclusion criteria were (1) previous treatment history for exudative AMD, including IRI or photodynamic therapy; (2) eyes that had undergone vitrectomy, (3) eyes with retinal angiomatous proliferation (4) other macular abnormalities including the myopic choroidal neovascularization, angioid streaks and other secondary CNV. If both eyes were eligible, the second eye was included in this study.

### Follow up

All patients received 3-monthly IAIs (2.0 mg/0.05 ml) followed by monthly follow-up requiring SD-OCT examination, as well as routine ophthalmic examination. The vertical and horizontal scans with 100 times averaging were obtained though the center of the fovea at each visit

Retreatment was determined on the basis of ophthalmoloscopy and OCT findings. Additional IAI was performed if intraretinal or subretinal fluid was seen on SD-OCT or new macular hemorrhage was seen on ophthalmoloscopy.

### Genotyping

We genotyped two single nucleotide polymorphisms (SNPs) including rs800292 in the *CFH* gene, and rs10490924 in the *ARMS2* gene. Peripheral blood was collected from the study participants. Genomic DNA was purified using a PUREGENE DNA Isolation Kit (Gentra Systems, Minneapolis, MN). Genotyping was performed using TaqMan genotyping assays with a 7300/7500 Real-Time PCR System (Applied Biosystems, Foster City, CA) in accordance with the manufacturer’s recommendations as we recently described^30^.

### Statistical analysis

Statistical analyses were performed using DR.SPSS for Windows (SPSS, Tokyo, Japan). BCVA measured in the decimal scale with Landolt chart was converted into a logarithm of the minimal angle resolution (log MAR). To convert from decimal scales to log MAR scales, we used the following formula “Decimal VA = 10^(−log MAR)^”. The differences between continuous variables and categorical variables were tested by Mann-Whitney U test, Kruskle-Wallis test or chi-square test. The paired t-test was used to determine the significance of difference between values before and after treatments. Multivariate logistic regression analysis was performed to investigate the baseline risk factors for retreatment due to residual or recurrent exudation. Cox regression analysis was conducted to estimate the relative risk for retreatment. P-values less than 0.05 were considered statistically significant.

## Additional Information

**How to cite this article**: Kikushima, W. *et al*. Incidence and risk factors of retreatment after three-monthly aflibercept therapy for exudative age-related macular degeneration. *Sci. Rep.* **7**, 44020; doi: 10.1038/srep44020 (2017).

**Publisher's note:** Springer Nature remains neutral with regard to jurisdictional claims in published maps and institutional affiliations.

## Figures and Tables

**Table 1:** Clinical and genetic characteristics of the patients according to age-related macular degeneration subtypes.

	All patients (n = 140)	Typical neovascular AMD (n = 71)	PCV (n = 69)	P value
Age	75.9 ± 8.1	78.7 ± 7.4	72.9 ± 7.9	4.2 × 10^−5^*
Male gender	101（72.1%）	42 (65.6%)	47 (83.9%)	0.27**
Baseline log MAR BCVA	0.45 ± 0.39	0.53 ± 0.41	0.36 ± 0.35	1.6 × 10^−3^*
Greatest linear dimension (μm)	3368 ± 1869	3250 ± 1559	3489 ± 2147	0.98*
Central macular thickness (μm)	417 ± 183	452 ± 204	381 ± 152	0.025*
Subfoveal choroidal thickness (μm)	213.8 ± 89.4	197.8 ± 78.6	230.3 ± 97.1	0.087*
*ARMS2* A69S (rs10490924)				
TT	56 (40.0%)	33 (46.5%)	23 (33.3%)	
TG	58 (41.4%)	29 (40.8%)	29 (42.0%)	
GG	26 (18.6%)	9 (12.7%)	17 (24.7%)	
T-allele frequency	60.7%	66.9%	54.4%	0.032**
*CFH* I62V (rs800292)				
GG	81 (57.9%)	47 (66.2%)	34 (49.3%)	
GA	50 (35.7%)	21 (29.6%)	29 (42.0%)	
AA	9 (6.4%)	3 (4.2%)	6 (8.7%)	
G-allele frequency	75.7%	81.0%	70.3%	0.037**

ARMS: age-related maculopathy susceptibility, BCVA: best-corrected visual acuity.

CFH: complement factor H, GLD: greatest linear dimension, log MAR: logarithm of the minimal angle resolution, BCVA: best-corrected visual acuity.

^*^Mann-Whitney U test.

^**^Chi-square test.

**Table 2:** Change of variables after initial intravitreal aflibercept injection.

	Baseline	3 M	6 M	9 M	12 M
Exudative AMD (n = 140)					
BCVA	0.45 ± 0.39	0.31 ± 0.30	0.28 ± 0.31	0.26 ± 0.31	0.26 ± 0.33
p-value	NA	1.9 × 10^−9^	2.7 × 10^−11^	4.0 × 10^−11^	4.1 × 10^−11^
Central macular thickness	417 ± 183	230 ± 116	248 ± 126	246 ± 108	242 ± 108
p-value	NA	4.9 × 10^−28^	6.1 × 10^−24^	7.9 × 10^−20^	4.6 × 10^−23^
Subfoveal choroidal thickness	214 ± 89	184 ± 82	187 ± 83	187 ± 83	187 ± 83
p-value	NA	2.9 × 10^−26^	6.4 × 10^−23^	6.8 × 10^−19^	9.2 × 10^−21^
PCV (n = 69)					
BCVA	0.36 ± 0.35	0.25 ± 0.28	0.21 ± 0.29	0.20 ± 0.30	0.19 ± 0.28
p-value	NA	3.1 × 10^−5^	1.1 × 10^−7^	1.3 × 10^−6^	4.7 × 10^−7^
Central macular thickness	381 ± 152	199 ± 78	224 ± 103	219 ± 84	220 ± 93
p-value	NA	6.9 × 10^−17^	2.7 × 10^−13^	1.1 × 10^−13^	3.3 × 10^−14^
Subfoveal choroidal thickness	230 ± 97	200 ± 90	207 ± 93	206 ± 90	205 ± 94
p-value	NA	1.9 × 10^−13^	4.8 × 10^−11^	7.0 × 10^−10^	3.2 × 10^−11^
Typical neovascular AMD (n = 71)					
VA	0.53 ± 0.41	0.36 ± 0.32	0.34 ± 0.32	0.32 ± 0.32	0.32 ± 0.35
p-value	NA	1.1 × 10^−5^	7.3 × 10^−6^	3.4 × 10^−6^	7.9 × 10^−6^
Central macular thickness	452 ± 204	259 ± 138	271 ± 142	271 ± 123	264 ± 118
p-value	NA	2.5 × 10^−13^	2.3 × 10^−12^	3.6 × 10^−9^	2.7 × 10^−11^
Subfoveal choroidal thickness	198 ± 79	168 ± 70	167 ± 68	169 ± 72	169 ± 68
p-value	NA	3.9 × 10^−14^	2.2 × 10^−13^	1.8 × 10^−10^	5.3 × 10^−11^

AMD: age-related macular degeneration, PCV: polypoidal choroidal vasculopathy, VA: visual acuity, NA: not available.

Paired t-test was performed for all statistical analyses.

**Table 3:** Baseline factors associated with BCVA and visual gain at 12 months.

Variables	BCVA at 12 months		Visual gain at 12 month	
	β-coefficient	p-value	β-coefficient	p-value
Age	0.064	0.38	0.0067	0.38
Male gender	−0.01	0.86	−0.013	0.86
Baseline BCVA (log MAR)	0.62	3.6 × 10^−14^	−0.59	2.1 × 10^−12^
AMD subtype (Typical neovascular AMD:0, PCV:1)	−0.027	0.73	−0.028	0.73
Central macular thickness (μm)	−0.088	0.21	−0.092	0.21
Subfoveal choroidal thickness (μm)	−0.14	0.039	−0.15	0.039
Greatest linear dimension (μm)	−0.027	0.70	−0.028	0.70
*ARMS2* A69S T allele	0.029	0.67	0.030	0.67
*CFH* I62V G allele	0.027	0.70	0.028	0.70

BCVA: best-corrected visual acuity, Log MAR: logarithm minimal angle of resolution, AMD: age-related macular degeneration, PCV: polypoidal choroidal vasculopathy, *ARMS*: age-related maculopathy susceptibility, *CFH*: complement factor H.

**Table 4:** Clinical and genetic factors associated with retreatment after 3 monthly intravitreal injections of aflibercept.

	Without retreatment (n = 46)	With retreatment (n = 94)	Univariate p-value	Multivariate p-value	Odds ratio	95% confidence interval
Exudative AMD (n = 140)						
Age	73.1 ± 7.3	77.2 ± 8.2	3.1 × 10^−3^*	7.2 × 10^−3^	1.08	1.02–1.14
Male gender	32 (69.6%)	69 (73.4%)	0.63**	0.37	1.52	0.61–3.78
PCV subtype	26 (56.5%)	43 (45.7%)	0.23**	0.73	1.17	0.48–2.88
Baseline log MAR BCVA	0.44 ± 0.39	0.45 ± 0.39	0.77*	0.26	0.52	0.17–1.63
Greatest linear dimension (μm)	3147 ± 2255	3476 ± 1650	0.049*	0.50	1.0	1.0–1.0
Central macular thickness (μm)	400 ± 189	425 ± 180	0.26*	0.41	1.0	1.0–1.0
Subfoveal choroidal thickness (μm)	237 ± 90	202 ± 87	0.036*	0.25	1.0	1.0–1.0
*ARMS2* A69S (rs10490924) T-allele frequency (TT:TG:GG)	45.7% (11:20:15)	68.1% (45:38:11)	2.7 × 10^−4^**	1.9 × 10^−3^	2.46	1.39–4.35
*CFH* I62V (rs800292) G-allele frequency (GG:GA:AA)	70.7% (24:17:5)	78.2% (57:33:4)	0.17**	0.37	1.35	0.70–2.61
	Without retreatment (n = 20)	With retreatment (n = 51)	Univariate p-value	Multivariate p-value	Odds ratio	95% confidence interval
Typical neovascular AMD (n = 71)						
Age	75.9 ± 5.9	79.7 ± 7.7	0.02*	0.15	1.08	0.97–1.19
Male gender	12 (60%)	33 (64.7%)	0.71	0.21	2.36	0.62–8.95
Baseline log MAR BCVA	0.49 ± 0.42	0.54 ± 0.40	0.41*	0.83	0.83	0.16–4.37
Greatest linear dimension (μm)	2334 ± 1272	3610 ± 1523	1.5 × 10^−3^*	0.047	1.001	1.000–1.001
Central macular thickness (μm)	415 ± 191	466 ± 208	0.34*	0.66	1.001	0.997–1.005
Subfoveal choroidal thickness (μm)	218 ± 66	190 ± 82	0.13*	0.19	0.994	0.986–1.003
*ARMS2* A69S (rs10490924) T-allele frequency (TT:TG:GG)	52.5% (6:9:5)	72.6% (27:20:4)	0.022**	0.15	1.97	0.77–5.10
*CFH* I62V (rs800292) G-allele frequency (GG:GA:AA)	75% (11:8:1)	83.4% (36:13:2)	0.26**	0.63	1.29	0.46–3.60
	Without retreatment (n = 26)	With retreatment (n = 43)	Univariate p-value	Multivariate p-value	Odds ratio	95% confidence interval
PCV (n = 69)						
Age	70.9 ± 7.6	74.2 ± 7.9	0.092*	0.083	1.08	0.99–1.17
Male gender	20 (76.9%)	36 (83.7%)	0.43	0.89	1.11	0.25–5.0
Baseline log MAR BCVA	0.40 ± 0.37	0.34 ± 0.35	0.39*	0.25	0.35	0.06–2.1
Greatest linear dimension (μm)	3773 ± 2643	3316 ± 1795	0.77*	0.57	1.00	1.00–1.00
Central macular thickness (μm)	388 ± 189	377 ± 127	0.69*	0.97	1.00	0.996–1.004
Subfoveal choroidal thickness (μm)	252 ± 103	217 ± 92	0.18*	0.37	0.997	0.991–1.003
*ARMS2* A69S (rs10490924) T-allele frequency (TT:TG:GG)	40.4% (5:11:10)	62.8% (18:18:7)	0.010**	0.02	2.49	1.15–5.39
*CFH* I62V (rs800292) G-allele frequency (GG:GA:AA)	67.3% (13:9:4)	72.1% (21:20:2)	0.55**	0.45	1.42	0.57–3.45

BCVA: best-corrected visual acuity, *ARMS*: age-related maculopathy susceptibility, *CFH*: complement factor H, GLD: greatest linear dimension, log MAR: logarithm of the minimal angle resolution, PCV: polypoidal choroidal vasculopathy.

^*^Mann-Whitney U test.

^**^Chi-square test.

**Table 5:** Cox regression analysis associated with retreatment-free period.

Variables	β-coefficient	p-value	Hazard ratio	95% confidence interval
Age	0.040	1.0 × 10^−2^	1.04	1.01–1.07
Male gender	0.21	0.40	1.23	0.76–1.99
Baseline BCVA (log MAR)	−0.16	0.59	0.85	0.47–1.53
AMD subtype (Typical neovascular AMD:0 PCV:1)	0.020	0.94	1.02	0.64–1.64
Central macular thickness (μm)	3.1 × 10^−4^	0.57	1.0	1.0–1.0
Subfoveal choroidal thickness (μm)	−1.2 × 10^−3^	0.39	1.0	1.0–1.0
Greatest linear dimension (μm)	2.1 × 10^−5^	0.73	1.0	1.0–1.0
*ARMS2* A69S T-allele frequency	0.42	6.0 × 10^−3^	1.53	1.13–2.07
*CFH* I62V G-allele frequency	0.088	0.64	1.09	0.76–1.58

BCVA: best-corrected visual acuity, Log MAR: logarithm of the minimal angle resolution, AMD: age-related macular degeneration, PCV: polypoidal choroidal vasculopathy, *ARMS*: age-related maculopathy susceptibility, *CFH*: complement factor H.

**Table 6:** Baseline BCVA and the changes from baseline in eyes with or without retreatment with additional intravitreal injections of aflibercept.

	Baseline BCVA	Visual acuity change from baseline			
		3 months	6 months	9 months	12 months
With retreatment (n = 94)	0.45 ± 0.39	−0.14 ± 0.25	−0.16 ± 0.27	−0.17 ± 0.30	−0.17 ± 0.31
Without retreatment (n = 46)	0.44 ± 0.39	−0.15 ± 0.29	−0.19 ± 0.29	−0.22 ± 0.30	−0.24 ± 0.32
p-value	0.77	0.89	0.40	0.31	0.23

BCVA: best-corrected visual acuity.
